# Parasites and Their Heterophagic Appetite for Disease

**DOI:** 10.1371/journal.ppat.1004803

**Published:** 2015-05-21

**Authors:** Vern B. Carruthers

**Affiliations:** Department of Microbiology and Immunology, University of Michigan Medical School, Ann Arbor, Michigan, United States of America; University of Wisconsin Medical School, UNITED STATES

## What Is Heterophagy?

Heterophagy (literally meaning “other eat”) is the process of a cell consuming material from its environment. Heterophagy involves vesicular uptake and thus is distinct from cellular acquisition of small metabolites by solute transport, which typically occurs via membrane channels or transporters in a way that is akin to sipping or siphoning from the environment. Endocytosis, macropinocytosis, trogocytosis, and phagocytosis are forms of heterophagy that contribute distinctly to ingestion of substances ranging from soluble macromolecules to insoluble substances, including other cells. Ingestion is typically coupled with digestion by hydrolytic enzymes in the endolysosomal system of the cell. Although less commonly used than its counterpart autophagy (“self eat”), the term heterophagy describes a distinct process that is rarely discussed in a cohesive manner and yet is often crucial for cell survival. Because heterophagy encompasses vesicular transport, this process is largely confined to eukaryotes. Consistent with the scope of *PLOS Pathogens*, this article will focus principally on eukaryotic pathogens, especially protozoan parasites.

## What Does Heterophagy Do?

Eukaryotic pathogens use several different pathways for heterophagy. Intestinal amoebic parasites including *Entamoeba histolytica* depend on phagocytic engulfment of large entities, including whole cells, to activate virulence, evade the immune system, disseminate, and obtain resources for replication [1]. The parasite causes tissue destruction by triggering apoptotic death of cells in the large intestine, which in some cases is followed by phagocytosis of the dying cell [2,3]. Recent findings suggest that *E*. *histolytica* internalization of bits of the target cell by trogocytosis elevates calcium in the target cell prior to cell death [4], implicating this form of cellular nibbling in the targeted destruction of cells and tissues. Cell killing via trogocytosis likely aids tissue invasion by *E*. *histolytica* and dissemination from the large intestine to other organs, including the liver, where severe disease transpires. Trogocytosis, along with receptor-mediated phagocytosis and digestion of erythrocytes, likely also helps satisfy the parasite’s appetite, thereby fueling rapid replication.

Kinetoplastid parasites such as *Leishmania* and African trypanosomes utilize prolific receptor-mediated endocytosis in a confined, harbor-like flagellar pocket that provides a sheltered site for receptor-mediated endocytosis of essential nutrients. Receptor-mediated endocytosis in trypanosomes facilitates the acquisition of iron-laden proteins including transferrin and hemoglobin [5,6] along with cholesterol and phospholipids associated with lipoproteins [6–8]. Parasite endolysosomal cathepsin proteases subsequently degrade the endocytosed proteins to liberate iron, cholesterol, and phospholipids for anabolic construction of daughter parasites during cell division. Receptor-mediated endocytosis of lipoproteins is also important for replication of intestinal *Giardia* parasites [9]. Thus, extracellular parasites utilize various forms of heterophagy to support parasite replication and activate virulence.

Intracellular parasites also employ heterophagy inside infected host cells. For example, the apicomplexan parasite *Plasmodium falciparum* and its brethren use a form of endocytosis for ingestion of hemoglobin and other proteins from the cytosol of infected erythrocytes. Proteolysis of hemoglobin within the parasite lysosome, termed the food vacuole, liberates amino acids to support parasite replication and create space for the parasite to grow within the infected cell. Unlike heterophagy by extracellular parasites, specific receptors for hemoglobin uptake by malaria parasites have not been identified, and electron microscopy studies suggest that uptake occurs mainly by nonspecific bulk flow [10–12]. The importance of this pathway is underscored by its vulnerability to many of the available antimalarial treatments, as further discussed below. Similar to malaria parasites, a second apicomplexan parasite, *Toxoplasma gondii*, ingests and digests proteins from the cytosol of the infected cell to support its intracellular replication [13]. This pathway also involves bulk flow since it is unlikely that the parasite has a specific receptor for the fluorescent proteins used to identify the pathway. Nonetheless, the involvement of specific receptors that target discrete host-derived proteins cannot be ruled out. It also remains to be seen the extent that other apicomplexan parasites besides those closely related to *Plasmodium* and *Toxoplasma* utilize heterophagy during intracellular replication.

## Why Is It Important for Parasites to Eat a Balanced Diet?

Several studies suggest that parasites satisfy nutritional requirements from the environment by utilizing both solute transport and heterophagic mechanisms. *P*. *falciparum* obtains methionine, isoleucine, or leucine [14] and likely other amino acids via transporters at the plasma membrane. On the other hand, all of the 20 amino acids except for isoleucine can be obtained via endocytosis and digestion of hemoglobin from infected erythrocytes. Remarkably, *P*. *falciparum* grows almost as fast in medium containing isoleucine as the sole amino acid as it does in medium replete with amino acids [15], suggesting that heterophagy is sufficient to satisfy nearly all of the parasite’s amino acid diet. Moreover, disrupting hemoglobin digestion substantially arrests parasite growth in medium containing all 20 amino acids [15], indicating a crucial role for heterophagy in *P*. *falciparum* replication. Interestingly, amino acid transporters on the parasite surface function most efficiently when exchanging one amino acid for another. Thus, partnership between transport and heterophagic pathways is suggested by the ability to import key exogenous amino acids such as isoleucine in exchange for surplus amino acids originating from the digestion of hemoglobin [14]. Although the relative contributions of transport versus heterophagy during the natural course of blood infection remain to be determined, these findings suggest that the parasite uses an exquisite collaboration between transport and heterophagy to satisfy its nutritional demands.

The relative roles of transport versus heterophagy in other parasites are less well described. *T*. *gondii* internalizes and degrades proteins from the cytosol of the infected cell. Since this parasite infects nucleated cells containing a wide repertoire of cytosolic proteins, its protein diet is likely much more diverse than that of *P*. *falciparum*. Although partially blocking degradation of host-derived proteins reduces *T*. *gondii* replication by ~30% in rich medium, the extent that this parasite utilizes heterophagy to supplement its protein diet is unknown.

## Are There Other Roles for Heterophagy?

As mentioned above, ingestion of certain bacteria by *E*. *histolytica* activates virulence in pathogenic strains. Interestingly, this parasite expresses a G-protein coupled receptor that binds lipopolysaccharide (LPS) from gram-negative bacteria [16], rendering the possibility of virulence activation via receptor-mediated signaling. *E*. *histolytica* exposure to *Escherichia coli* increases its phagocytic and cytolytic activity and up-regulates expression of parasite cysteine proteases, which contribute to virulence by degrading target cells and inactivating components of the host immune response [17,18]. Thus, although the mechanistic details are yet to emerge, *E*. *histolytica* might use heterophagy as a sensory pathway to up-regulate virulence factors that elevate its utilization of environmental resources, simultaneously increasing its ability to cause disease.

*T*. *gondii* strains deficient in the proteolytic degradation of host proteins obtained by heterophagy are virulence attenuated in mice and have a 60%–80% lower parasite burden 4 days post-infection with low or medium parasite inocula [13]. This decrease is more pronounced than expected from the 30%–40% reduction in parasite replication by such strains in culture. Interestingly, mice infected with high inocula of wild-type and protease-deficient parasites show similar parasite burden through the course of infection. Mice lacking immune activation by the cytokine interferon gamma also show comparable parasite loads for wild-type and protease-deficient parasites. Together, these results suggest that the decrease in parasite burden is not entirely due to intrinsic slower replication of the protease-deficient strain and that heterophagy might also contribute to evasion of host immunity.

## Is Heterophagy a Susceptible Target?

Although heterophagy is central to survival for many parasites, it can also be a liability. For example, certain African trypanosomes fail to infect humans because we produce specialized lipoproteins termed trypanosome lytic factors (TLFs) that enter the parasite, at least in part, via the same receptor-mediated endocytic pathway the parasite uses to obtain hemoglobin and lipoproteins [6]. TLF1 ruptures the parasite lysosome, causing parasite death. Remarkably, trypanosomes that cause human African sleeping sickness express specific proteins that either bind to and neutralize TLFs [19,20] or confer resistance by interacting with endocytic membranes [18]. The extent that these immune evasion proteins can be exploited for new therapies remains to be seen.

Malaria heterophagy of host hemoglobin is also a well-known Achilles heel of the parasite. Most antimalarial medicines kill the parasite by directly or indirectly interfering with the crystallization of toxic heme liberated by proteolysis of ingested hemoglobin in the parasite food vacuole [21]. However, like African trypanosomes, malaria parasites have evolved ways of minimizing the efficacy of antimalarial drugs, including most notably an ability to ship them out of the food vacuole [22]. Although attention has been mostly focused on events in the food vacuole itself, little is known about how hemoglobin is delivered to the food vacuole.

Overall, this article highlights the centrality of heterophagic pathways to parasite survival and pathogenesis. Deeper understanding of heterophagy is needed to better appreciate this fundamental aspect of parasite infection biology as well as to expose new targets for intervention.
